# Social Bacteriophages

**DOI:** 10.3390/microorganisms8040533

**Published:** 2020-04-07

**Authors:** Pilar Domingo-Calap, Lucas Mora-Quilis, Rafael Sanjuán

**Affiliations:** 1 Institute for Integrative Systems Biology, I^2^SysBio, Universitat de València-CSIC, 46980 Paterna, Spain; pilar.domingo@uv.es (P.D.-C.); lucas.mora@uv.es (L.M.-Q.); 2 Department of Genetics, Universitat de València, 46980 Paterna, Spain

**Keywords:** social evolution, sociovirology, cooperation, virus–virus interactions, bacteriophage

## Abstract

Despite their simplicity, viruses can display social-like interactions such as cooperation, communication, and cheating. Focusing on bacteriophages, here we review features including viral product sharing, cooperative evasion of antiviral defenses, prudent host exploitation, superinfection exclusion, and inter-phage peptide-mediated signaling. We argue that, in order to achieve a better understanding of these processes, their mechanisms of action need to be considered in the context of social evolution theory, paying special attention to key population-level factors such as genetic relatedness and spatial structure.

## 1. Introduction

In social organisms, individual traits can be influenced by interactions with other members of the population. Microorganisms, including bacteria and unicellular eukaryotes, are known to display many social characteristics [1]. However, these features remain relatively understudied in viruses. A major question is how, from a mechanistic point of view, viruses can interact socially, since many traits exhibited by complex organisms, such as individual recognition, are not displayed by viruses. Recent work has revealed novel aspects of virus–virus interactions, particularly in bacteriophages, of which we will provide specific examples in this review. However, in order to understand why these mechanisms exist, we need to contextualize them within existing social evolution theory [2]. Achieving this goal first requires elucidating the fitness implications of the interactions at play. For instance, imagine a population of cooperators that secrete a public good, defined as a useful resource available to others. If a variant that does not produce this resource appears in the population, it will benefit from the good without incurring production costs. Thus, everything else being equal, this selfish variant (or cheater) will be favored by selection, possibly leading to extinction of the cooperators. Hence, social features need to be investigated not only from a mechanistic approach, but also from a population-level perspective. The above example actually illustrates a central problem for the evolution of cooperation, namely that natural selection should in principle disfavor traits that diminish the actor´s fitness, such as altruism. Yet, since such traits do exist in nature, there must be factors that promote their evolutionary stability.

Generally speaking, cooperation can evolve if it preferentially benefits other cooperators, that is, if selfish individuals are excluded [1,3,4]. This general idea has been developed in different ways. For instance, Darwinian fitness can be redefined as a compound of direct and indirect effects (inclusive fitness). The direct effects of a trait are those experienced by the actor itself, whereas indirect effects are those experienced by others. Costly social traits might be favored by selection through indirect effects. One way of increasing indirect fitness is by directing cooperative or altruist actions towards genetically related individuals (kin selection). Even if viruses are not known to display kin recognition, assortment among cooperators can still be fostered by spatial population structure. Indeed, most viruses spread in the form of infection foci, which are typically constituted by the progeny of a single founder, meaning that interactions occur preferentially among closely related viruses. Recent work has shown that kin selection theory can be applied to viral populations [5].

Below we review several types of virus social interactions and discuss them from an evolutionary perspective. We focus on bacteriophages, which have been used as model systems in biology for a century and which have revealed recently new, unexpected features, such as inter-phage communication and altruistic evasion of hosts defenses. Yet, many of the discussed processes also apply to other types of viruses.

## 2. Sharing of Phage Products during Coinfections

As discussed above, variants that do not produce a certain public good take advantage of cooperators without reciprocating, promoting the loss of the public good. This type of outcome is generally referred to as the tragedy of the commons. Viruses coinfecting a given cell can share products such as capsids or other structural proteins, which therefore function as intracellular public goods. It has been amply shown that, under extensive coinfection regimes, defective viruses evolve readily in populations [6]. Such defective viruses have incomplete viral genomes missing essential genes and, thus, can be replicated and packaged only in cells coinfected with functional viruses, called helpers. Streamlined genomes are replicated faster, and defective viruses can even give up transcription, becoming purely passive replicators. For these reasons, defective viruses thriving at the expense of helpers can reach very high population frequencies when coinfections are abundant. One of the first studies that demonstrated this extreme form of cheating was conducted with coliphage f1 [7]. When the phage was serially passaged at high virus/cell ratios, which promoted extensive coinfection, rapid accumulation of defective mutants lacking more than 70% of the genome was observed.

**Figure 1:**
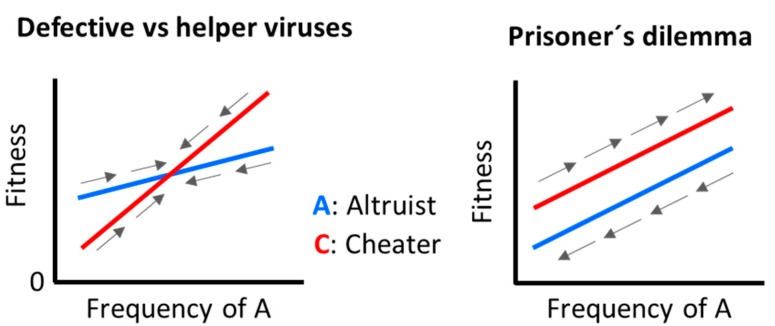
Two examples of altruistic-cheater virus interactions. Both defective-helper systems and Prisoner´s dilemma exhibit frequency-dependent selection, but they differ in the arrangement of fitness values, leading to different population dynamics. Defective viruses cannot reach fixation because they ultimately depend on helper variants. In contrast, in Prisoner´s dilemma, the cheater fully outcompetes the altruistic variant. Arrows indicate the direction of selection.

The relationship between coinfection levels and cheating was investigated in experimental populations of the *Pseudomonas* phage Ø6 [8]. The study focused on the interaction between two phage variants (here called A and C). At low phage/cell ratios, most cells were infected by a single phage type and hence interactions between the two variants were infrequent. This allowed measuring the fitness of A alone (*f_A|A_*) and of C alone (*f_C|C_*). In contrast, at high phage/cell ratios, mixed infections became frequent, which provided information about the fitness of each variant in the presence of the other (*f_A|C_* and *f_C|A_*). These measurements yielded the following arrangement of fitness values: *f_C|A_* > *f_A|A_* > *f_C|C_* > *f_A|C_*. Thus, A functioned as an altruistic virus, whereas C functioned as a cheater. Based on these measured values, C should be able to invade A populations, whereas A could not invade C populations. In mixed populations the cheater was systematically fitter than the altruist and, consequently, altruists should go extinct, despite the fact that a population made entirely of altruists would show the highest fitness. In game theory, this paradox is known as Prisoner´s dilemma, and its outcome is similar to the tragedy of the commons. As discussed above, cheater invasion can be prevented if cooperators interact preferentially with other cooperators, as was demonstrated in subsequent experiments with Ø6 [9]. However, it should be noted that the Ø6 cheaters were not defective viruses. With defective cheaters, the payoff matrix would be *f_C|A_* > *f_A|A_* > *f_A|C_* > *f_C|C_* = 0, meaning that C could never fully outcompete A. Hence, the interplay between helper and defective viruses is not an example of Prisoner´s dilemma (Figure 1). 

## 3. Phage Cooperation to Overcome CRISPR Immunity

**Figure 2:**
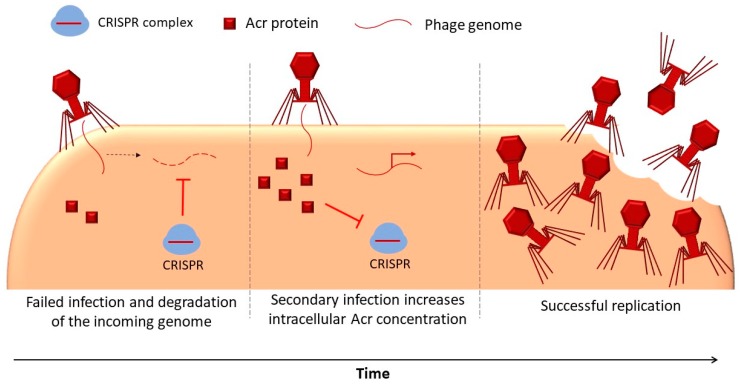
Anti-CRISPR proteins as an intracellular public good. A first (altruistic) virus expresses Acr proteins, but these are not sufficient to inactivate the CRISPR system, leading to degradation of the viral genome and abortive infection. However, the cell remains in a transient immunosuppressed state due to the action of the Acr proteins, which allows a second (potentially identical) virus to overcome CRISPR and successfully complete the infection.

The CRISPR-Cas system (clustered regularly interspaced short palindromic repeats and associated proteins) is a prokaryotic immune system of which the primary function is to counteract phage infections. Small sequences of a phage genome are incorporated into the CRISPR loci of the bacterial chromosome, and subsequently, expression of the derived CRISPR RNAs guides the targeting and sequence-specific destruction of new incoming phage genomes by host nucleases [10]. However, in their evolutionary arms race against bacteria, phages have incorporated anti-CRISPR (Acr) proteins [11]. The social evolution of Acr proteins has been investigated in DMS3m, a phage of *Pseudomonas aeruginosa* [12,13]. An interesting feature of Acr proteins is that they typically do not achieve full inactivation of CRISPR immunity. As a result, a single Acr-encoding phage is likely to not succeed in infecting its host and is often degraded. This abortive infection nevertheless debilitates the CRISPR system, making the pre-infected cell more susceptible to re-infections by Acr-encoding phages. Thus, the first, unsuccessful invader can be considered as an altruistic virus, whose degradation allows a second member of the population to succeed (Figure 2). Recently, it has been shown that Acr-negative variants can exploit CRISPR-debilitated cells to some extent, although they fail to take over the population because they get only a limited benefit from Acr-positive phages [14]. Based on this, it has been hypothesized that powerful Acr proteins may pay a cost in terms of invasion risks by Acr-negative cheaters, which could in turn explain why Acr proteins have not evolved greater potency. Acr proteins have also been revealed in phages infecting *Streptococcus* [15] and in archaeal viruses [16], which could allow for further testing of the above ideas.

## 4. Extracellular Phage Public Goods

Secreted public goods have been studied extensively in bacteria [1,17,18]. However, little is known about the production of extracellular public goods in viruses. An interesting, yet still poorly studied case is provided by phage depolymerases. Depolymerases are hydrolases capable of specifically digesting the exopolysaccharide (EPS) capsule of certain bacteria [19]. The EPS capsule is an important virulence factor that hampers immune recognition, but also serves as a barrier against phage infections [20]. Important pathogenic enterobacteria such as *Acinetobacter baumannii*, *Escherichia coli*, and *Klebsiella pneumoniae* produce EPS capsules. Phage depolymerases, which are required for infecting these bacteria, are usually anchored to spike or tail fiber proteins of the phage virion, albeit some are secreted as soluble diffusible proteins [21]. The latter may be considered as an extracellular public good, whereas anchored depolymerases could be viewed as privatized goods, but they might also be shareable in two ways. First, if multiple phages bind the same cell surface, the effects of their depolymerases might benefit them collectively. Second, upon lysis, cells release free phage tails in addition to complete virions. These tails could diffuse locally and digest EPS capsules for the benefit of other members of the population. Lysins [21], which degrade bacterial cell walls, could also potentially function as public goods, similarly to depolymerases.

**Figure 3:**
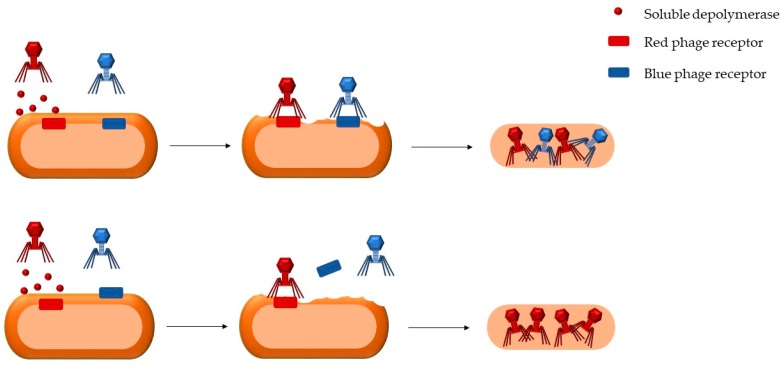
Hypothesized depolymerase-mediated phage-phage interactions. Soluble depolymerases should be shareable extracellular products allowing for synergistic or antagonistic interactions among phages. (**Top**) A depolymerase produced by the red phage digests the exopolysaccharide (EPS) capsule, exposing phage receptors attached to the cell membrane, but also exposing the blue receptor, which promotes entry of the blue phage. (**Bottom**) A depolymerase produced by the red phage digests the EPS capsule, exposing its receptor, but also releasing the blue receptor, which was embedded in the EPS capsule, thus blocking entrance of the blue phage.

Proof of principle for depolymerase-mediated cooperation was provided in a study involving two different species of coliphages [22]. One of the phages was strongly lytic, but had poor ability to penetrate the EPS. In contrast, the other phage was capable of depolymerizing EPS, but had a low lytic capacity. Coinfection with both phages resulted in a synergistic interaction whereby lysis and progeny production increased disproportionally. However, depolymerase-mediated phage–phage interactions need not to be mutually beneficial by definition. For instance, it is conceivable that digestion of the EPS capsule by a given depolymerase might remove the receptor of another phage, impeding its attachment (Figure 3).

## 5. Prudent Phages

Prudent exploitation of resources in a population is a cooperative action that increases long-term fitness at the expense of short-term fitness. As such, the evolution of prudent exploitation poses the same issues as the evolution of altruism. From the phage point of view, bacteria are exploitable resources, and if these resources are exhausted, the viral population will go extinct. To prevent this, phages can, for instance, reduce their infectivity, which will slow down phage population growth in the short term to ensure long-term survival. However, if the population also contains “rapacious” variants with higher infectivity, these will outcompete the prudent phages, leading to resource overexploitation and a long-term reduction of the mean population fitness. Whether this sort of tragedy of the commons can be avoided depends, to a large extent, on the spatial structure of the population [23]. In low-dispersion environments, where population structure is strong, interactions will preferentially take place among related individuals. Demes containing rapacious phages will initially growth fast, but will ultimately be outcompeted by other demes containing prudent phages. Hence, again cooperation will evolve if cooperators (here, prudent phages) interact preferentially with other cooperators. In this scenario, selection acts at two levels (intra- and inter-deme), and hence is referred to as multi-level selection, or group selection. Intra-deme selection favors rapacious phages, whereas inter-deme selection favors prudent phages. If demes are well-isolated, prudent variants will be favored by inter-deme selection, whereas if mixing occurs frequently, intra-deme selection, and thus rapacious phages, should prevail [24].

The genetic and mechanistic basis of prudent host exploitation was investigated using the ID11 coliphage [25]. Following experimental evolutionary passages in the presence of spatial structure, large lysis plaques appeared. Counterintuitively, this phenotype was achieved by reducing the efficiency of phage adsorption (without altering lysis time or burst size). This increased the duration of the infection cycle, providing more time for the susceptible host bacterial population to expand before being infected, which in turn increased the number of infections over the long term and led to improved phage spread. At the molecular level, the prudent phenotype was conferred by an amino acid in the major capsid protein. There are other examples of prudent phages, such as the case of *Pseudomonas fluorescens* phage Ø2 [26]. This study showed that, under limited resource availability (i.e., low bacterial density), rapacious phages prevailed in the population regardless of spatial structure, whereas spatial structure determined the evolution of prudency with high resource availability.

## 6. Communication among Phages

Many phages have facultative lytic and lysogenic cycles. The lytic cycle culminates with host lysis induced by phage proteins and release of progeny virions. In the lysogenic cycle, in contrast, phages integrate their genomes into the bacterial chromosome and remain as latent prophages that replicate together with their host. Lysogenic phages can undergo lysis following certain triggers, which can be complex and are subject to strong stochasticity [27]. Clearly, the lysis–lysogeny decision is critical for phage survival and represents a choice between two radically different infectious strategies. Recently, it has been discovered that phages of the spBeta group of *Bacillus* sp. encode a communication system, called "arbitrium", which regulates lysis-lysogeny decisions [28]. The system is based on secretion of a small peptide called AimP that allows sensing phage population density. Bacteria internalize AimP using a specific transporter, and the peptide is then recognized by a phage receptor. AimP suppresses the transcription of a negative lysogeny regulator, called AimX. When the phage population density increases, the AimP concentration also augments in the medium and AimX expression thus decreases, allowing lysogeny to occur. Conversely, when the phage population density is low, the AimP concentration decreases and bacterial lysis is consequently induced. Hence, the arbitrium system allows the phage to lyse the host specifically when competition for resources (hosts) is expected to be low. 

Arbitrium constitutes the first inter-phage communication system discovered, yet its function remains to be investigated from a social evolution perspective. In principle, a phage could produce (but not sense) AimP to suppress progeny production in competitors via lysis inhibition. However, such a phage would incur a cost in terms of lysing cells when resources are scarce, which may prevent progeny virions from finding susceptible host cells. What has been shown is that AimP peptides have diverged rapidly between species, and that structural changes in the receptors can help increase signal specificity [29]. This suggests that cross-signaling is detrimental either for the producers or the receptors of the signal.

## 7. Superinfection Exclusion

Superinfection exclusion is a process whereby a virus resident in a cell blocks infection with additional viruses. The mechanisms of action are varied and include removal of the receptor, reduction of particle internalization, or blocking at the level of replication or transcription. Superinfection exclusion was first described in bacteriophages, but occurs in all kinds of viruses [30]. It operates not only in lytic phages, but also in lysogenic phages. Indeed, there is strong pressure for lysogenic phages to avoid superinfection, since entry of a lytic phage will result in bacterial lysis and loss of the prophage. In *Pseudomonas aeruginosa*, prophages can block superinfection by several mechanisms including modifications of surface antigens such as type IV pilus or the O-antigen [31]. Another example is provided by *Streptococcus thermophilus*, in which prophages mediate superinfection exclusion via the Ltp lipoprotein encoded in the lysogenic module. Ltp is anchored to the external cell membrane, inhibiting the injection of incoming phage genomes [32]. Recent work has also used RNA-seq to identify new antisense RNAs that mediate superinfection exclusion in the *Salmonella* phage BTP1 [33].

**Figure 4:**
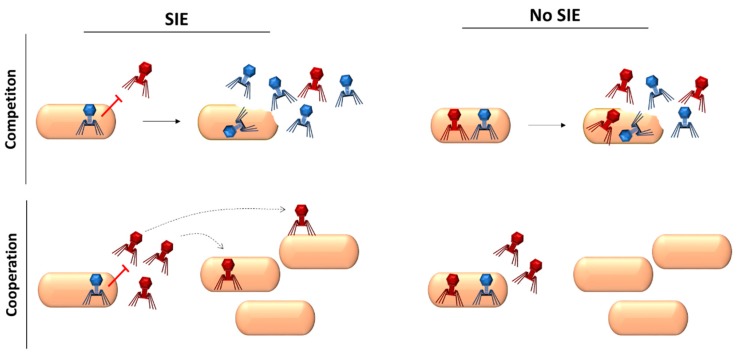
Two possible evolutionary interpretations of superinfection exclusion (SIE). Superinfection exclusion could be considered as a mechanism for avoiding competition for intracellular sources. In this scenario, superinfection exclusion would directly increase the fitness of the actor virus, which would capitalize on cellular resources at the expense of the excluded virus. Alternatively, superinfection exclusion could be considered as a cooperative mechanism whereby the actor virus would pay the cost of promoting the spread of the excluded virus (indicated by dotted arrows) to uninfected neighbor cells, which would improve overall resource exploitation.

The evolutionary significance of superinfection exclusion remains obscure in most cases. A null hypothesis is that exclusion needs not to be an adaptive process, but could simply be a byproduct of the extensive changes experienced by the infected cell. However, since some phages (and other viruses) have specific genes that control superinfection exclusion, it is reasonable to postulate that this may be a selectively advantageous trait. The most intuitive evolutionary explanation for superinfection exclusion is that it allows the actor virus to prevent competition for resources exerted by incoming viruses. Yet, it is also possible that superinfection exclusion may function as a cooperative trait that benefits the excluded virions by preventing them from entering already infected cells and allowing them to search for alternative cells containing more available resources (Figure 4). This was indeed postulated in a study with vaccinia virus, in which virus-encoded proteins were shown to mediate repulsion of virions from the surface of the infected cell, which could subsequently reach other, uninfected cells, speeding up viral spread [34]. Under the cooperation hypothesis, the actor virus would pay the cost of actively preventing superinfection and of helping other members of the population reach susceptible cells that would otherwise be available to the actor´s progeny. Hence, superinfection exclusion would be favored by selection only if the excluded viruses shared high genetic relatedness with the actor (cooperator assortment). Interestingly, superinfection exclusion is known to work best among highly related viruses. 

## 8. Phage Aggregation and Collective Spread

Collective infectious units are structures that mediate the joint transmission of multiple viral genomes at the intercellular and/or interhost levels. These include polyploid capsids, virion aggregates, and extracellular vesicles containing pools of virions, among others [35]. By promoting cellular coinfection with multiple genomes, collective infectious units favor virus–virus interactions, which can be cooperative or non-cooperative. It has been long postulated that different genetic variants of a virus that coinfect a cell can interact in a mutualistic way by complementing their specific genetic defects, or by combining their gene products in a way that promotes new functions [36,37]. Additionally, invading cells with multiple viral particles at once may boost the initial stages of infection and promote successful establishment of the infection, akin to an Allee effect [38], particularly in difficult-to-infect cells that deploy strong antiviral responses [39]. On the other hand, by increasing coinfection levels, collective spread may favor the emergence of cheater variants such as defective viruses, unless the co-transmitted genomes share high levels of genetic relatedness [40,41,42]. 

In phages, collective spread can take place when multiple copies of the phage genomes are jointly encapsidated (polyphage), as shown in the filamentous coliphage f1 [43]. Also, changes in pH, temperature, or ionic strength in the environment can favor virion aggregation [44], as has been observed in the coliphages ØX174 [45], MS2 [46], and T4 [47]. Yet, the evolutionary implications of collective spread have been poorly studied in phages. A scenario in which increasing the number of viral genome copies transmitted to a given cell might prove beneficial for the phage is counteraction of CRISPR-Cas immunity. As discussed above, Acr proteins typically achieve only partial CRISPR inactivation, and reinfection is often required to tip the balance in favor of the phage. Hence, it can be speculated that collective spread might help phages combine their Acr proteins and increase the likelihood of overcoming CRISPR defenses, although this remains to be shown. 

## 9. Conclusions

Despite pioneer studies and recent advances, social virus interactions remain underexplored. In some cases, mechanisms enabling such interactions (e.g., arbitrium, aggregation, proteins mediating superinfection exclusion) have been characterized in some detail, yet the basic social evolution aspects of these traits remain to be investigated. In some other cases (e.g., Ø6 Prisoner´s dilemma, prudent phages), social processes have been characterized well enough for allowing a quantitative assessment of their evolution, but the mechanisms of action need to be revealed. A combined approach, in which mechanistic considerations provide information about possible evolutionary outcomes, and population-level analysis helps us explain why and how these mechanisms have evolved, would provide a more comprehensive view of virus biology. Understanding the social nature of viruses might also prove useful for practical applications in biomedicine or biotechnology. For instance, cheater invasion should be considered in protocols aimed at improving a given product through directed evolution techniques. Also, the virulence of a given pathogen is related to the evolution of prudent exploitation strategies, allowing us to establish predictions that link transmission modes with disease severity. Another possible application involves phage therapy, which typically relies on phage cocktails for increasing efficacy, preventing resistance, or broadening the spectrum of action of a phage product. However, cocktails should provide a fertile ground for phage–phage interactions, which need to be investigated to assess their implications for treatment efficacy.
